# Microplastics in Persian Gulf offshore sediments: Distribution, polymer composition, and ecological risk assessment

**DOI:** 10.1016/j.isci.2026.117263

**Published:** 2026-08-28

**Authors:** Parisa Amiri, Afsaneh Shahbazi, Ali Mehdinia

**Affiliations:** 1 Environmental Sciences Research Institute, Shahid Beheshti University, Tehran, Iran; 2 Iranian National Institute for Oceanography and Atmospheric Science, Tehran, Iran

**Keywords:** microplastics, Persian Gulf, offshore sediments, ecological risk

## Abstract

This study assesses microplastics (MPs) pollution and ecological risk in offshore sediments of the Persian Gulf, a strategic semi-enclosed sea with limited offshore MPs data. To address this gap, we integrate the pollution load index (PLI), polymer hazard index (PHI), and potential ecological risk index (PERI), using Raman-validated polymer identification to link MPs abundance to polymer-specific hazardousness. Sediments from 39 stations contained 5–45 particles kg^−1^, indicating broad anthropogenic influence rather than localized sources. Black fibers dominated, and polyethylene terephthalate (PET) was the most frequent polymer. However, high-hazard polymers (polyvinyl chloride and polystyrene) primarily drove risk, decoupling ecological risk from particle abundance. Although most stations had low PLI values, T1S3, in the northwestern Persian Gulf, was consistently identified as a critical risk zone by PHI and PERI because of hazardous-polymer accumulation. These findings show that polymer-level toxicity is a more sensitive indicator of offshore ecological risk than abundance alone.

## Highlights

• First Persian Gulf offshore microplastic risk assessment using PHI, PLI, and PERI indices
• Station T1S3 was the highest-risk zone due to its toxic polymers
• PET is the main polymer (41%), mostly black (67%) and fiber-shaped (74%) microplastics
• Localized hotspots (e.g., station T1S3) exhibited extreme ecological risk based on results

## Graphical abstract

**Figure:**
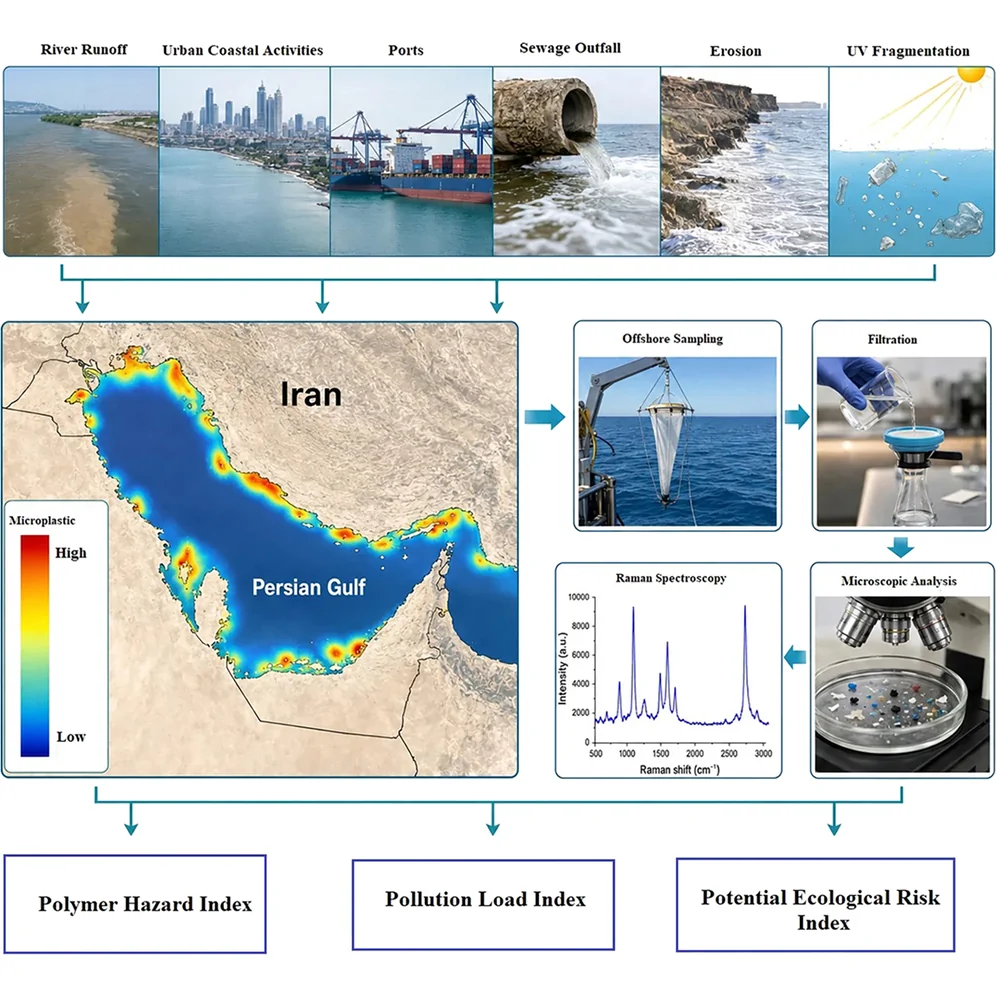

## Teaser

Environmental science; Environmental issues; Applied sciences

## Introduction

Plastics have increasingly emerged as attractive substitutes for traditional materials in industrial settings, largely due to their reduced weight and the potential economic advantages they offer during periods of operational downtime.^1^ Projections indicate that global plastic production could surpass 600 million metric tons by 2050, reflecting a near doubling of existing output levels.^2^ In the Persian Gulf, countries of the Gulf Cooperation Council (GCC) collectively produce more than 30 million tons of plastics annually, while Iran contributes approximately 5 million tons.^3^ The accelerating growth of plastic production and consumption is intensifying plastic waste generation, amplifying its environmental footprint in marine systems.^4^^,^^5^ Recent global estimates suggest that cumulative plastic leakage into the oceans could reach 250 million metric tons by 2040 without transformative mitigation.^6^ Microplastics (MPs <5 mm) have emerged as widespread and persistent pollutants in aquatic environments.^7^ Mismanaged plastic waste enters marine systems each year, where physical, chemical, and biological processes gradually fragment macroplastics into MPs.^8^ More than 400,000 tons of plastic debris are estimated to enter the Persian Gulf annually.^1^ The Gulf’s semi-enclosed morphology, shallow bathymetry, restricted water circulation, and high evaporation together extend the residence time of pollutants, making the basin notably susceptible to long-term MPs accumulation.^9^ Distinct thermohaline patterns in the Gulf may influence the transport of these particles from coastal zones toward offshore sinks.^10^ Once in marine environments, MPs undergo fragmentation via UV radiation, mechanical abrasion, and microbial action, which increase their surface area and environmental reactivity.^11^^,^^12^ This enhanced reactivity promotes sorption of persistent organic pollutants (POPs), heavy metals, and polycyclic aromatic hydrocarbons (PAHs), enabling MPs to act as vectors for hazardous substances.^13^^,^^14^ Experimental studies have shown that MPs may induce oxidative stress and physiological disturbances in marine organisms.^15^ Thus, the ecological risks posed by MPs result from both their physical presence and their capacity to participate in chemical and biological interactions. The Persian Gulf is a globally strategic marine ecosystem, supporting major fisheries, offshore oil and gas platforms, desalination plants, and rapidly expanding coastal populations. Anthropogenic pressures-offshore hydrocarbon extraction, dense maritime traffic, brine discharge, and municipal-industrial effluents combined with natural hydrodynamic constraints, intensify pollutant accumulation in sediments.^16^^,^^17^ Sediments function as long-term sinks for MPs, integrating local discharge and regional transport mechanisms.^18^ However, their potential to act as secondary sources of pollution remains a concern for the Gulf’s fragile biodiversity.^3^ Despite the global focus on MPs, offshore sediments of the Persian Gulf have received little attention. Most previous studies concentrate on beaches, surface waters, or nearshore sediments; as a result, basin-wide distributions, polymer composition, and ecological risk in offshore zones remain poorly constrained.^10^ This knowledge deficit limits regional risk modeling and hampers effective management approaches. Furthermore, many Persian Gulf and international studies emphasize MPs abundance, while often neglecting polymer-specific toxicity, which critically influences ecological hazard potential.^10^^,^^19^ This oversight is particularly significant in the Persian Gulf, a semi-enclosed basin under intense industrialization and maritime activity, with diverse polymer types introduced by petrochemical industries, each exhibiting distinct hazard profiles.^11^ Given the region’s unique combination of industrial pressures and restricted circulation, identifying polymer-specific risks is particularly essential. Despite the increase in research on MPs in the Persian Gulf and similar systems, several methodological constraints hinder a comprehensive risk assessment. Many studies rely mainly on visual sorting or density-based extraction, often without systematic spectroscopic validation, leading to possible misidentification of particles and inaccurate polymer characterization. Even when spectroscopic methods (e.g., Fourier transform infrared [FTIR]) are used, identifying weathered, pigmented, or biofouled particles in complex matrices can remain challenging. Moreover, the main research focus has been on coastal hotspots, while offshore depositional environments, key sinks for MPs accumulation are underexplored, which restricts basin-scale understanding. Similarly, ecological risk assessments frequently utilize abundance-based indicators, often overlooking the fundamental role of polymer-specific hazards.

To address these limitations, the present study provides a comprehensive evaluation of MPs pollution in offshore sediments of the Persian Gulf by employing high-resolution Raman spectroscopy and a multi-index risk assessment framework. Raman spectroscopy provides reliable identification of polymers through their vibrational fingerprints and facilitates detection of dark, weathered, or morphologically altered particles not easily classified with conventional methods. Ecological risk was assessed using three complementary indices: the pollution load index (PLI), the polymer hazard index (PHI), and the potential ecological risk index (PERI), collectively capturing contamination magnitude, polymer-specific toxicity, and integrated ecological risk. By combining spectroscopic confirmation with a toxicity-informed and multi-index ecological risk assessment, this work delivers a comprehensive baseline for offshore ecological risk from MPs in the Persian Gulf, providing a mechanistic framework for understanding the environmental implications of MPs pollution in marine sediments.^15^^,^^19^ This integrated framework strengthens the scientific basis for future environmental monitoring and risk mitigation in the region. Here, it was assumed that the ecological risk associated with MPs in the studied aquatic system is largely governed by the combined influence of polymer composition and particle abundance, reflecting variations in dominant input sources. Specifically, sites receiving higher contributions from high-toxicity or degradation-resistant polymer types, together with elevated MPs concentrations, were expected to exhibit proportionally greater PLI-, PHI-, and PERI-based ecological risk scores.

## Results and discussion

### Statistical analysis and distribution of MPs

**Figure 1:**
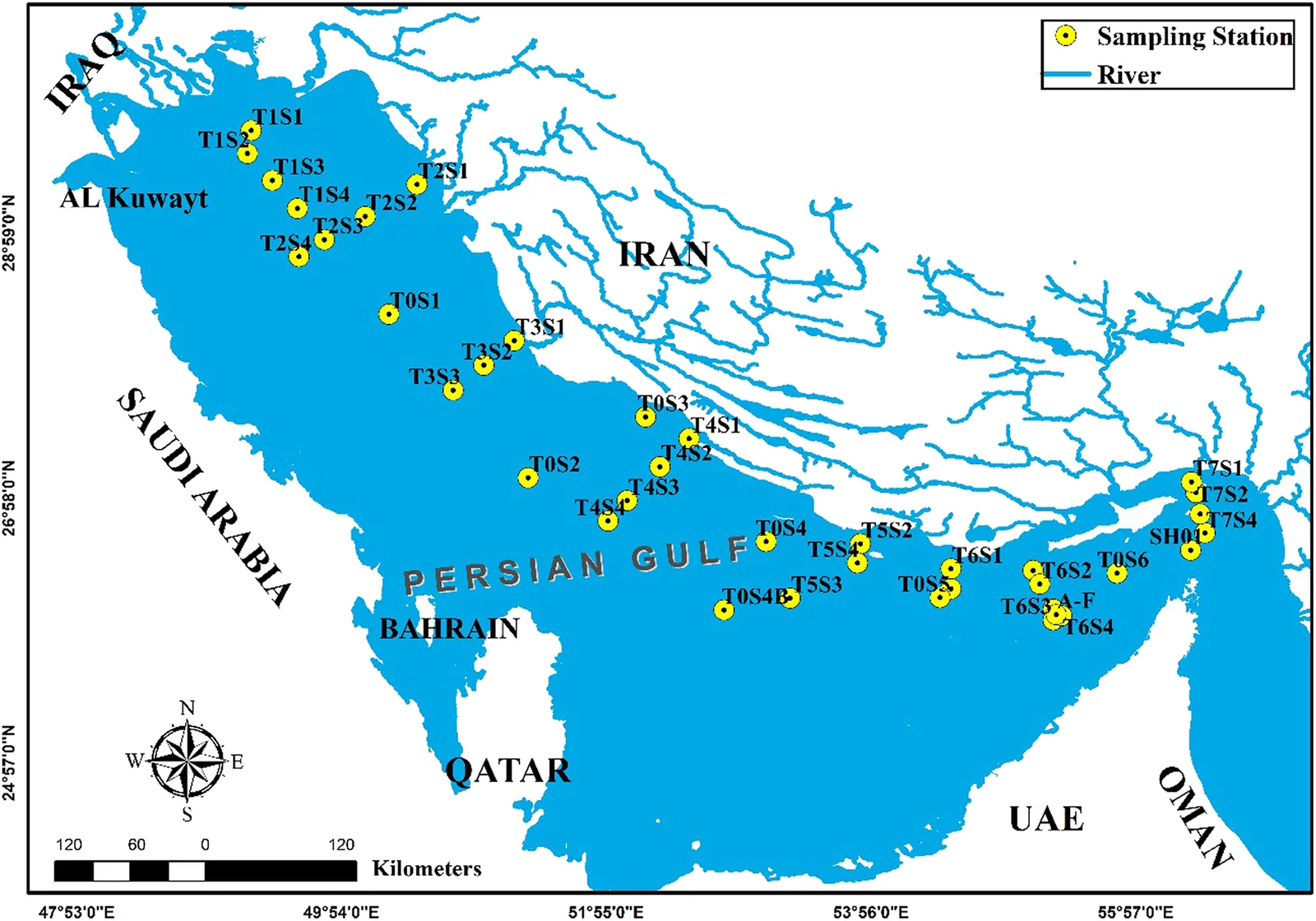
Locations of sediment sampling stations in the Persian Gulf

**Figure 2:**
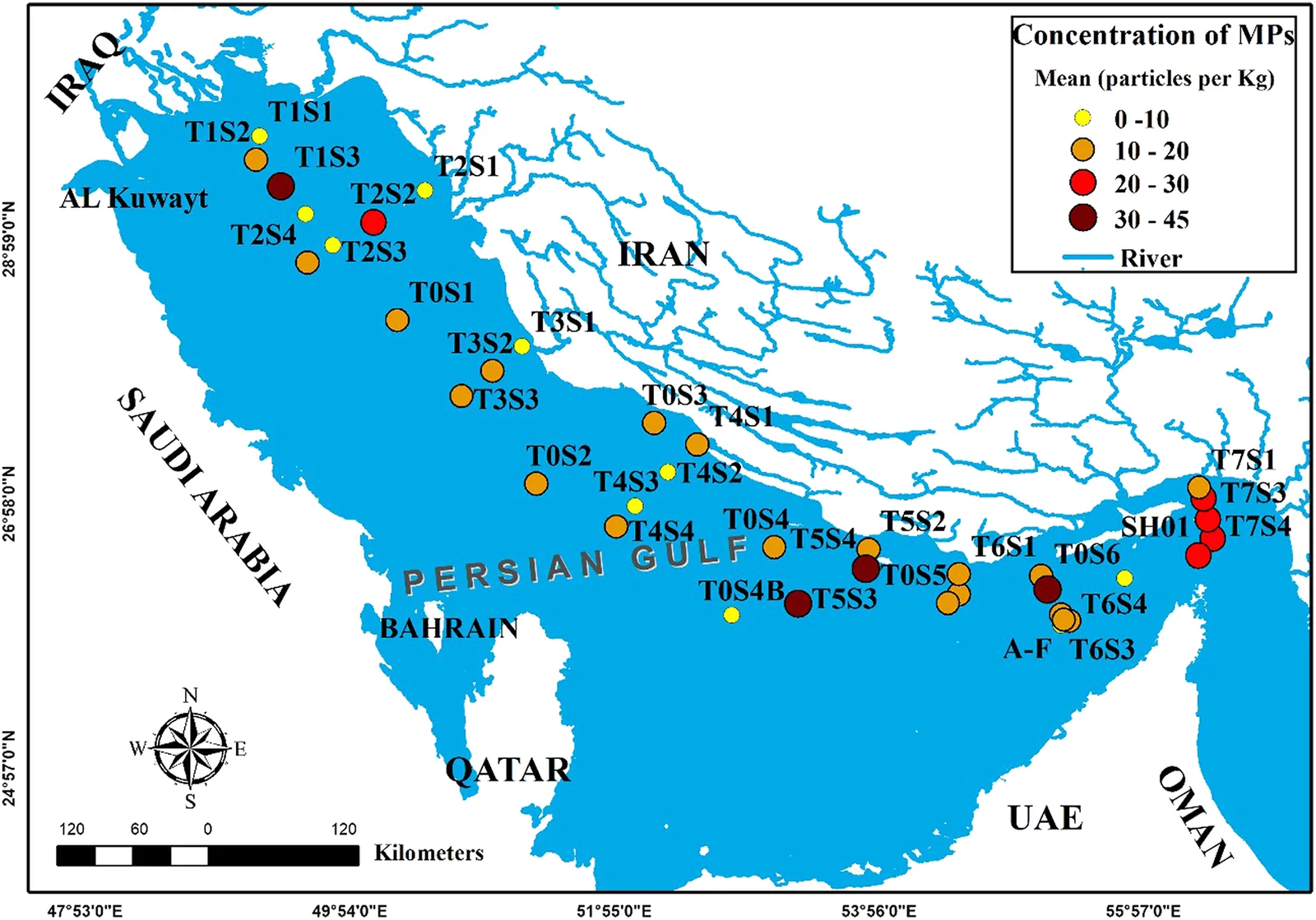
Concentration of microplastics in the Persian Gulf sediments

Descriptive statistical analyses were performed to evaluate the distribution patterns of MPs concentrations across the sampling stations (Figure 1, Table S1). The Shapiro-Wilk test was applied to assess the normality of the data. In five stations, *p* values were below 0.05, indicating non-normal, right-skewed distributions, which are typical for environmental MP datasets. Accordingly, the Kruskal-Wallis test, a nonparametric alternative to ANOVA, was employed to determine whether significant differences existed among stations, yielding a highly significant result (Sig = 0.00, *p* < 0.05). Post hoc pairwise comparisons were conducted using Mann-Whitney U tests with adjusted significance thresholds to control type I error. The use of non-parametric statistical methods is appropriate due to the non-normal, zero-inflated, and often skewed nature of environmental MPs data.^20^ MPs concentrations in Persian Gulf sediments ranged from 5 to 45 particles/kg, with higher levels observed near industrial zones and shipping corridors, and lower concentrations in less-impacted regions. Although pollutant sources were not directly traced in this study, this pattern may indicate a possible influence of anthropogenic activities on the spatial distribution of MPs. These patterns are consistent with global observations showing MPs even in remote marine environments.^18^ The spatial distribution of MPs is heavily influenced by hydrodynamic processes, especially tidal currents, which regulate accumulation and redistribution of particulate pollutants.^17^ MPs frequently act as vectors for hazardous substances, including POPs, heavy metals, and emerging contaminants, creating considerable ecological risks.^21^ A detailed concentration map (Figure 2) highlighted MPs pollution hotspots driven by anthropogenic activities and hydrodynamic forces. Notably, no MPs were detected at four stations (T1S4, T2S3, T4S2, and T4S3). This may indicate relatively lower anthropogenic influence at these sites; however, the absence of detected particles should be interpreted cautiously given methodological constraints inherent to MPs analysis. Additionally, recent studies have emphasized that methodological discrepancies, such as differences between net-based and bulk-sediment sampling, can influence MPs detection rates in the Persian Gulf. This highlights the need for cautious interpretation when comparing across studies.^22^^,^^23^

### Particle size distribution of MPs

The size distribution of MPs in offshore sediments of the Persian Gulf provides insight into prevailing degradation processes and environmental conditions. In the present study, the 500–1000 μm (30%) and 1000–1500 μm (29%) size classes were dominant, jointly representing 59% of the identified particles (Figures S1 and S1.1). Approximately 11% of particles fell within the 100–500 μm range, whereas particles larger than 2,000 μm accounted for about 14%. No MPs particles smaller than 100 μm or larger than 3,000 μm were reliably confirmed within the analytical framework of this study. However, particles smaller than 100 μm could occasionally be observed during microscopic examination but they were not reliably confirmed as MPs and were therefore excluded from the final Raman-based polymer identification. Although MPs in the 100–500 μm range constituted a relatively small proportion of the total, their ecological relevance remains considerable due to their enhanced bioavailability and greater potential for ingestion by marine organisms, thereby facilitating trophic transfer and bioaccumulation.^24^^,^^25^ The comparatively low abundance of particles larger than 2,000 μm may be associated with fragmentation processes under intense hydrodynamic activity, high solar radiation, and elevated temperatures characteristic of the Persian Gulf, which can accelerate the breakdown of larger plastic debris into smaller particles.^26^

### Color distribution of MPs

The color composition analysis of MPs in offshore sediments from the Persian Gulf (Figures S2 and S2.2) revealed a distinct distribution pattern. Black-colored particles were among the dominant types observed. Similar particles have occasionally been reported in association with tire wear,^27^ industrial coatings, and petroleum-related activities in coastal and industrial environments. However, color alone cannot be considered a reliable indicator of source. The coloration of MPs may arise from manufacturing additives, environmental weathering processes such as photo-oxidation,^28^ or the adsorption of organic and inorganic substances during environmental exposure, including biofilm formation^29^ that can darken originally lighter plastics. Therefore, any interpretation regarding potential sources in the present study should be regarded as tentative, as no direct source-tracing analyses (e.g., pyrolysis, gas chromatography-mass spectrometry [GC-MS], or specific chemical markers) were performed. Consequently, these observations are presented only as possible indications of anthropogenic influence rather than confirmed source attributions. Current evidence is insufficient to clearly distinguish between inherently dark particles and those that have darkened through environmental alteration in this region. Yellow (16%), blue (8%), and white (6%) MPs, often reported in connection with single-use plastics and municipal waste,^30^ were also observed. Red particles (3%) were the least abundant and have been associated in previous studies with specialized industrial applications, although pigment fading under UV exposure may also influence their environmental persistence.^31^ Hydrodynamic sorting and sediment deposition processes may further influence color distributions indirectly through size- and density-dependent transport mechanisms. From an ecotoxicological perspective, black particles may contribute to localized thermal stress due to their higher solar absorption capacity,^7^ while yellow and blue particles may pose increased ingestion risk because their coloration can resemble prey items for certain fish species.^32^ Additionally, the association of MPs abundance with elevated phthalate and organic pollutant levels^33^ highlights their potential role as vectors for hydrophobic contaminants. Despite extensive documentation of color alterations in MPs, the specific environmental drivers of particle darkening in the Persian Gulf remain insufficiently understood.

### MPs shape and polymer composition

Morphological analysis revealed that fibers constitute the predominant MPs type in the offshore sediments of the Persian Gulf, accounting for 74% of the total identified particles (Figures S3 and S3.1). Fragments and films represent secondary categories, comprising 23% and 3%, respectively.^34^ The dominance of fibrous MPs is primarily attributed to multiple anthropogenic sources, including the widespread release of synthetic fibers from textile materials, degradation of fishing gear, and domestic laundry wastewater discharge.^35^ To address potential airborne fiber contamination, rigorous quality assurance/quality control (QA/QC) procedures were implemented throughout the analytical workflow. These measures included the use of cotton laboratory coats, covered glassware, and procedural blanks processed alongside the samples. Analysis of procedural blanks revealed negligible fiber counts, indicating that laboratory contamination was minimal and that most detected fibers likely originated from environmental sources.

Particularly high concentrations were documented at station T1S3 near the confluence of the Arvand, Hendijan, and Karun rivers with marine waters. This spatial pattern likely reflects the combined influence of riverine inputs and regional anthropogenic activities. However, this interpretation should be considered cautiously and does not imply direct causal attribution.^36^^,^^37^ The ecological significance of this morphological distribution is substantial, as fibrous MPs demonstrate high bioavailability and an increased likelihood of ingestion across trophic levels, potentially facilitating toxic substance transfer through marine food webs.^25^ Concurrently, the presence of secondary fragments indicates the continued breakdown of larger plastic debris, reflecting persistent challenges associated with waste management in the region.^38^

Comprehensive polymer analysis identified polyethylene terephthalate (PET) as the dominant polymer (41%), followed by polyethylene (PE) at 23% and polypropylene (PP) at 15%, polyvinyl chloride (PVC) at 10%, polyamide (PA) at 6%, and polystyrene (PS) at 5% (Figures S4 and S4.1). This composition profile is consistent with recent investigations in the Persian Gulf^33^ and with earlier reports of PET predominance in regional sediments.^39^ Polymer identification was verified using Raman spectroscopy supported by reference spectral libraries, and a spectral match quality threshold of ≥0.80 was applied to minimize misclassification.

The relatively high density of PET (1.38–1.41 g/cm^3^) compared to seawater promotes its deposition and accumulation in benthic sediments.^40^^,^^41^ In addition, biofouling processes may increase the effective density of initially buoyant polymers such as PE and PP, thereby contributing to their eventual deposition in sediments.^42^ These physical characteristics partly explain the predominance of PET in sediments and illustrate how polymer properties influence environmental distribution. From an ecotoxicological perspective, PET is considered a relatively high-hazard polymer due to its environmental persistence, pollutant adsorption capacity, and potential for additive leaching.^19^ PE and PP, classified as medium-hazard polymers, nevertheless demonstrate a significant capacity for adsorbing hydrophobic contaminants and releasing potentially toxic additives under environmental conditions.^13^^,^^43^ PET accounted for the largest proportion of the identified polymers (41%). The predominance of PET observed in this study is broadly consistent with polymer composition patterns reported from marine sediments in the Persian Gulf and other coastal environments.^33^^,^^39^^,^^44^ Nevertheless, regional comparisons should be interpreted cautiously, as differences in sampling design, sediment characteristics, and analytical detection limits may influence reported polymer distributions.^45^

The relatively high proportion of PET (41%) observed in this study is comparable to values reported in several semi-enclosed marine systems, although methodological variability among studies should be considered when making regional comparisons.^46^ This pattern may partly reflect regional consumption trends, particularly the widespread use of bottled beverages, together with limitations in waste-management infrastructure. However, direct source apportionment was beyond the scope of the present study and would require targeted forensic polymer analyses. The spatial distribution of MPs in the Persian Gulf likely reflects the combined influence of multiple interacting factors.^47^ Anthropogenic inputs, including untreated sewage discharge, urban runoff, and economic activities such as oil industry operations, agriculture, fishing, and shipping, represent important potential sources.^34^ In addition, hydrodynamic processes such as current patterns, tidal fluctuations, and wave action influence MPs transport and deposition.^48^ These processes, together with particle density, morphology, and size, may affect burial efficiency and long-term sediment accumulation.

Consequently, sediments in the Persian Gulf may function as an important sink for MPs, while episodic disturbances such as storms or dredging may remobilize buried particles and act as secondary sources.^49^ These findings highlight the need for coordinated regional monitoring programs and the adoption of standardized analytical frameworks consistent with current UNEP (United Nations Environment Programme) recommendations.^46^

### Polymer Hazard Index (PHI) MPs

**Figure 3:**
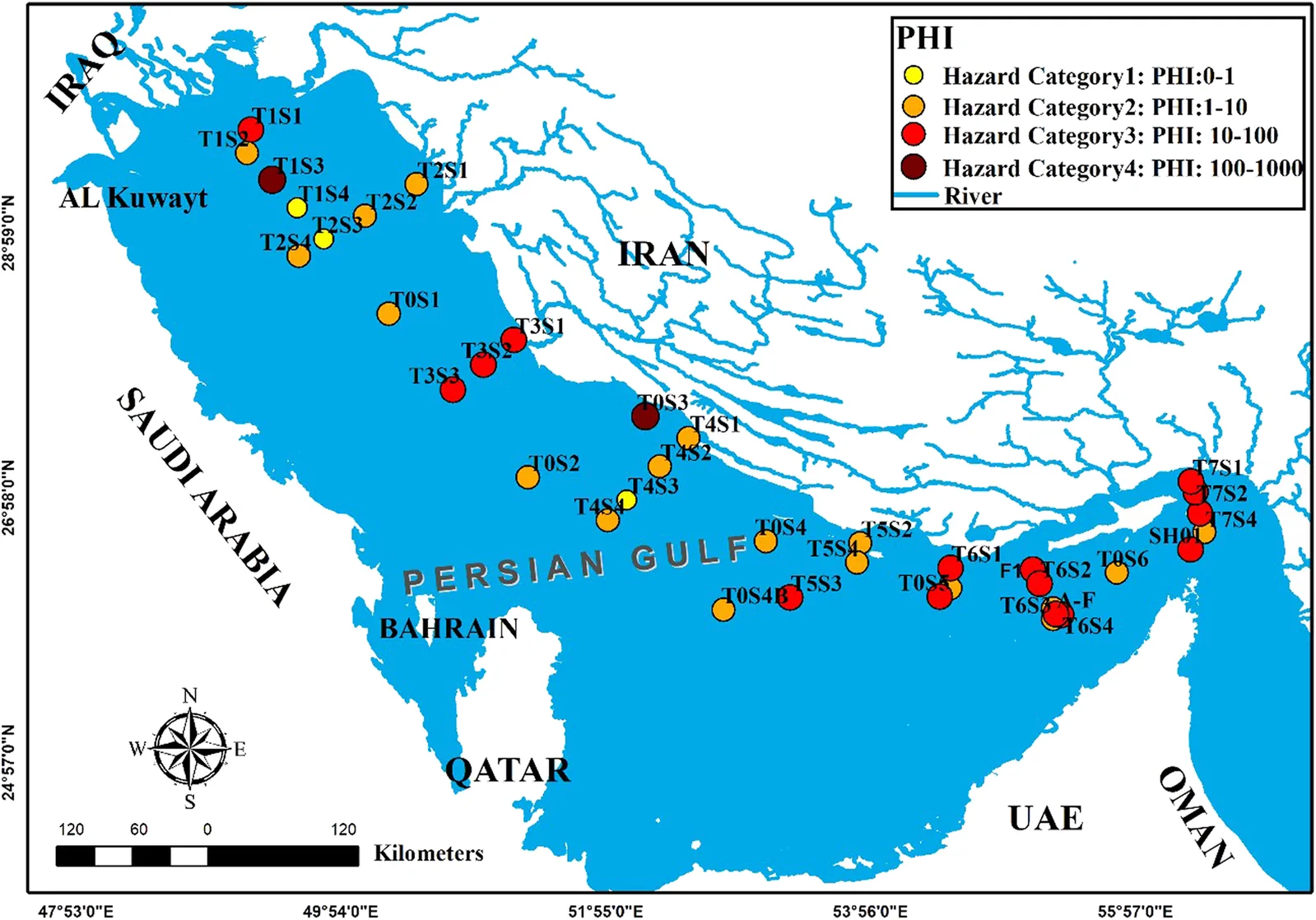
Polymer hazard index of Persian Gulf sediments

The PHI serves as a quantitative tool for assessing the integrated ecological hazard posed by MPs in marine sediments, incorporating particle concentration, polymer type, and physicochemical properties.^19^ However, PHI represents a relative hazard metric rather than a direct measure of biological exposure, and its interpretation therefore requires caution in ecological assessments.^50^ In this study, PHI is applied solely as a comparative indicator of polymer-specific hazard rather than a predictor of ecological impact. Analysis of the 39 sampling stations revealed substantial spatial heterogeneity in PHI values across the Persian Gulf (Figure 3). Stations were classified into very high (PHI = 100–1000), high (10–100), moderate (1–10), and low (<1) hazard categories. Two stations fell within the very high category, with station T1S3 exhibiting the maximum PHI value, followed by T0S3. The elevated PHI values at these locations were primarily associated with polymers assigned high hazard ratings.^19^^,^^51^ Although these polymers were not dominant in overall abundance, their hazard weighting within the PHI calculation resulted in a disproportionate contribution to the final index values. Distinct spatial patterns were observed, with higher PHI values concentrated in the northwestern part of the Gulf. While several major rivers (e.g., Arvand, Karun, and Hendijan) enter this region, the available dataset does not support attribution of observed PHI patterns to specific riverine inputs. These hydrological systems are therefore discussed only as potential regional-scale influences rather than confirmed sources.^36^^,^^37^ Similar river-associated accumulation trends have been reported in other semi-enclosed basins,^26^ but the present results do not verify such mechanisms in the Persian Gulf. Nine stations were categorized as high hazard, four as moderate, and the remainder as low hazard. Elevated PHI values were more frequently observed in coastal areas; however, potential contributions from industrial complexes, petrochemical activity, port operations, or fisheries cannot be confirmed based on the current dataset. These factors are therefore discussed only as general possible contributors, consistent with global trends reported in the literature.^52^ Interpretation of PHI as an indicator of specific pollution sources is not recommended without supplementary source-tracking analyses such as additive fingerprinting or degradation-pattern profiling.^49^ Environmental characteristics of the Persian Gulf may further shape the spatial distribution of PHI. The Gulf’s semi-enclosed configuration and long residence time (3–5 years) facilitate the retention of buoyant pollutants.^53^ These interpretations rely on established hydrodynamic descriptions in the literature, as no hydrodynamic measurements or modeling were conducted in the present study. Fine-grained, organic-rich sediments can enhance MPs retention,^54^ but sedimentological parameters were not measured here and are therefore considered only as potential contextual factors. A gradient from higher-hazard coastal areas to lower-hazard offshore regions was evident. Although similar patterns have been reported in other semi-enclosed seas,^35^ the absence of hydrodynamic or sediment-transport data limits the strength of this interpretation. In summary, the PHI assessment reveals pronounced spatial variability across the study area, with localized hotspots driven primarily by the presence of polymers assigned higher hazard categories. The findings indicate spatially elevated relative hazard but do not imply exceptional contamination levels when compared with other marine systems. PHI values should thus be interpreted as relative indicators of polymer-associated hazard rather than direct measures of toxicological risk. Additional investigations involving hydrodynamic modeling, finer spatial resolution, biological exposure studies, and advanced source-tracking approaches are necessary to establish more definitive conclusions regarding the origin and ecological implications of MPs pollution in the region.

### Pollution Load Index (PLI) of MPs

**Figure 4:**
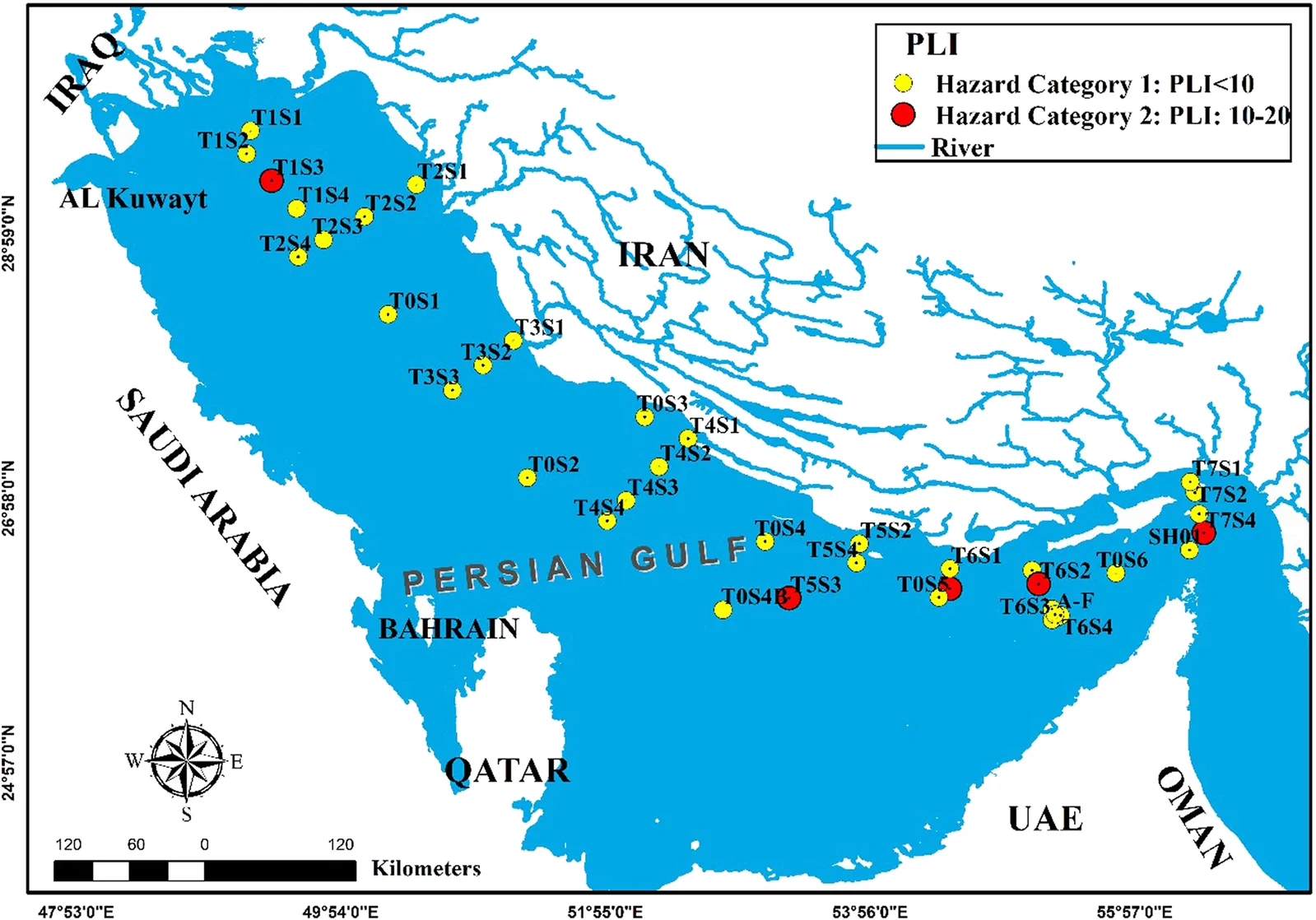
Pollution load index of Persian Gulf sediments

The PLI was employed to evaluate the integrated level of MPs contamination across the offshore sediments by comparing detected abundances with baseline values (Figure 4). In the absence of validated pre-industrial background levels, the minimum observed MPs concentration in the present dataset was adopted as the baseline (C_oi_) for PLI calculation. This pragmatic approach is consistent with established methodologies in MPs research.^48^^,^^55^ Notably, this baseline aligns with the minimum concentrations reported in other sediment-based assessments across the Persian Gulf,^39^ including the most recent study in this specific regional setting.^56^ Such consistency between independent regional datasets reinforces the use of the lowest measured abundance as an operational proxy for background conditions, ensuring that the PLI provides a comparative assessment of relative anthropogenic loading while reducing the likelihood of overestimating pollution severity. The majority of offshore sampling stations were assigned to category 1 (PLI <10), indicating that MPs loads remain relatively low across large sectors of the Persian Gulf’s offshore environment. This suggests that at these distances from the coast, hydrodynamic dispersion and the settling rates of larger particles (>100 μm) may limit significant accumulation.^39^ However, as noted by recent methodological assessments, these low PLI values should be interpreted with caution, as they may reflect the exclusion of particles smaller than 100 μm, which often constitute a significant fraction of the total plastic load.^57^^,^^58^ Figure 4 highlights several critical hotspots classified as category 2 (PLI 10–100), where localized pressures on the benthic ecosystem are evident. Station T1S3, located in the northwestern sector exhibited a high PLI. This pattern may be consistent with potential inputs from the nearby Arvand, Karun, and Hendijan river systems; however, no hydrodynamic modeling or source-tracing analyses were conducted in the present study to substantiate this interpretation. These river systems serve as major conduits for urban and industrial plastic waste from highly populated upstream basins.^37^ The spatial clustering of category 2 stations in the eastern sector, specifically stations T5S3, T6S2, and T7S2 near Bandar Lengeh and the Strait of Hormuz, points toward a plausible association with intense shipping traffic, port activities, and untreated municipal runoff from densely populated coastal hubs.^40^^,^^59^ The spatial variation in PLI values reflects a complex interplay between anthropogenic input and regional hydrodynamics. The anti-cyclonic circulation patterns and seasonal currents in the Persian Gulf likely facilitate the transport of MPs from coastal point sources toward specific offshore sinks.^53^ While the overall offshore PLI is low, the presence of category 2 hotspots suggests that certain areas act as accumulation zones due to reduced current velocities or specific sediment trap characteristics.^54^ Compared to other semi-enclosed basins like the Mediterranean or the Bohai Sea, the offshore PLI of the Persian Gulf appears moderate, yet the localized hotspots exceed levels reported for several open-sea environments.^60^ Ultimately, while the PLI provides a standardized metric for comparison, the reported values are inherently tied to the methodological detection limits. The absence of very small MPs in our dataset means the current PLI likely represents a conservative estimate of the actual pollution load.^49^ Future assessments incorporating sub-100 μm fractions and site-specific hydrodynamic modeling are essential to confirm these spatial trends and clarify the mechanisms of offshore MPs sequestration.

### Potential Ecological Risk Index (PERI) For MPs

**Figure 5:**
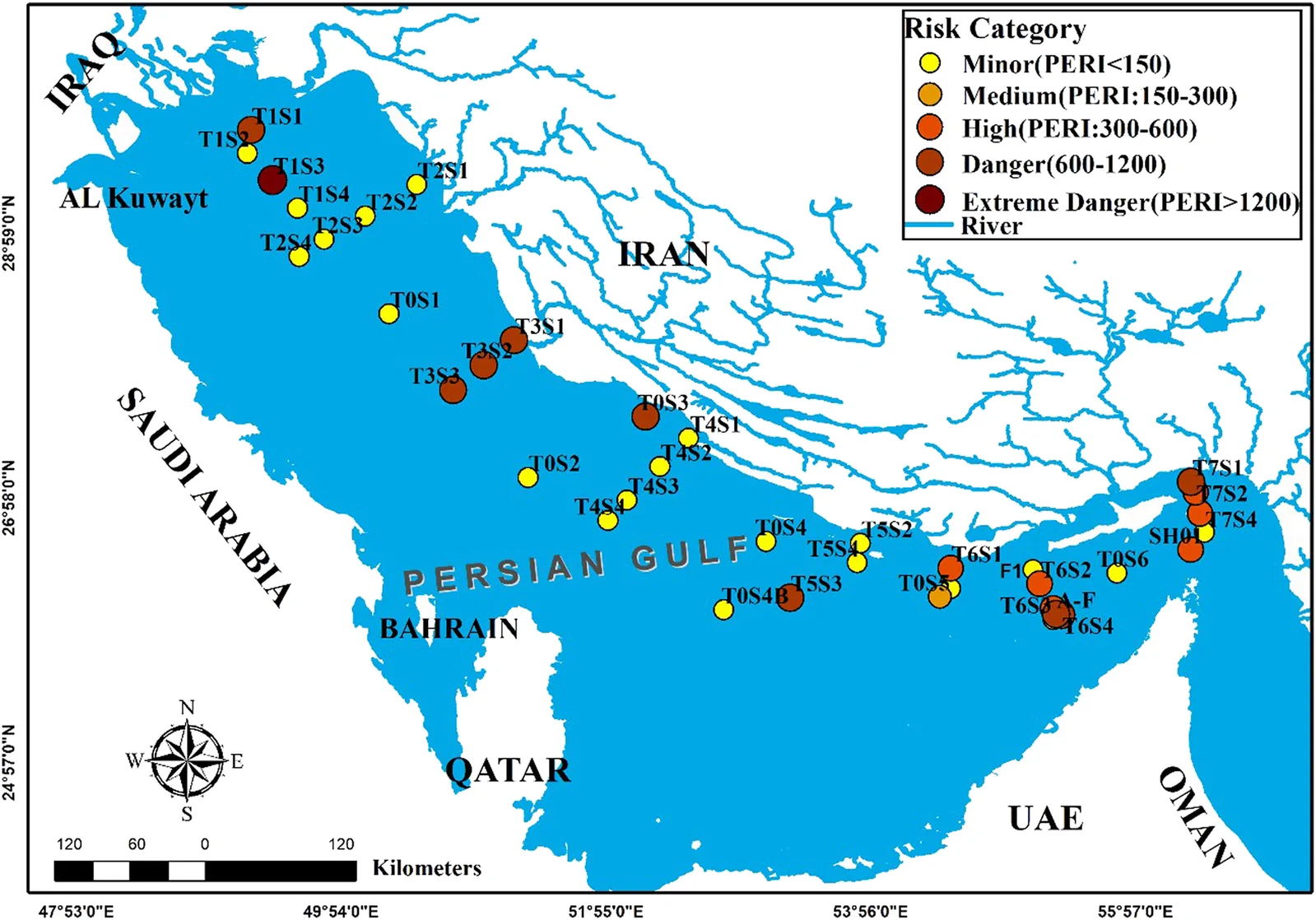
Potential ecological risk index in the Persian Gulf

The ecological risk assessment based on the PERI revealed substantial spatial heterogeneity across the offshore sediments of the Persian Gulf (Figure 5). The spatial map indicates that station T1S3 falls within the extreme danger category (PERI >1200), while nine stations including T1S1, T3S3, T3S2, T3S1, T0S3, T7S1, T5S3, T6S4, and A-F are classified as danger (PERI: 600–1200). This pattern highlights the presence of localized hotspots where the chemical toxicity of dominant polymers is likely to elevate ecological risks. At station T1S3, the dominant presence of PVC and PA is particularly noteworthy, as PVC contains chlorine-based additives and various stabilizers associated with high toxicity potential.^19^ Similarly, the stations categorized as high risk are characterized by elevated proportions of PA, a polymer known to release hazardous monomers and degradation by-products under marine conditions.^24^ Six stations T6S2, T5S5B, T7S3, T7S2, SH01, and T6S1, fell within the high or considerable risk classes, primarily associated with the presence of moderately toxic polymers such as PE and PP. Although PE and PP exhibit lower intrinsic toxicity than PVC and PS, their high hydrophobicity and capacity to adsorb POPs and heavy metals enable them to act as major vectors of secondary contaminants.^49^^,^^51^ Recent investigations further demonstrate that progressive fragmentation into particles <100 μm significantly increases their bioavailability, amplifying ecological exposure pathways.^58^ Stations categorized under moderate or minor risk appear to be dominated by lower-toxicity polymers such as PET, or contain comparatively low quantities of hazardous polymers. PET is broadly distributed in marine sediments due to its widespread use, yet demonstrates relatively limited capacity for adsorbing pollutants compared to PVC and PS.^4^^,^^52^ The spatial distribution illustrated in Figure 5 shows that the highest PERI levels (red markers) cluster around the northwestern and southeastern sectors of the Gulf. This pattern plausibly reflects the influence of major riverine inputs (e.g., the Arvand River system) in the northwest and intense maritime activities, petrochemical operations, and wastewater discharges near the Strait of Hormuz in the southeast.^23^^,^^59^ However, consistent with reviewer feedback, these associations are now presented as probabilistic rather than definitive, given the absence of direct source-tracing data. Importantly, the PERI values in this study should be interpreted with caution because the method relies heavily on polymer toxicity weighting and does not incorporate particle size dependent toxicity or additive-specific hazards.^49^ Furthermore, the exclusion of MPs <100 μm from our dataset may have led to an underestimation of ecological risk in stations dominated by polymers with high susceptibility to photo-oxidative chain scission and mechanical fragmentation particularly PE and PP. Ecologically, stations with high or very high PERI values may pose elevated ecological risk, potentially leading to biological impacts, such as gastrointestinal blockage, reduced feeding efficiency, oxidative stress, and reproductive impairment.^61^^,^^62^ The potential for MPs to adsorb hazardous chemicals further amplifies the risk of trophic transfer to higher organisms, including commercially important fish species.^40^^,^^63^ Overall, the PERI results underscore the central importance of polymer composition in shaping spatial risk patterns across the Gulf’s offshore sediments, while also highlighting the roles of proximity to pollution sources and hydrodynamic retention zones in generating localized hotspots. Future studies incorporating fine-fraction MPs (<100 μm), *in situ* chemical analyses of additives, and hydrodynamic modeling are essential to refine ecological risk estimates. In addition to these findings, it is important to place the applied risk assessment approach within the broader methodological context. These indices are used here as relative ecological risk indicators and should be interpreted within the limitations of a toxicity-weighted framework that does not fully capture all MPs-specific physical effects such as particle size, shape, aging, and polymer fragmentation dynamics.

### Limitations of the study

This study provides one of the first spatially explicit assessments of MPs contamination in offshore sediments of the Persian Gulf; however, the interpretation of its findings should be considered within the constraints of the applied methodological framework. In particular, MPs particles smaller than 100 μm could not be reliably confirmed within the applied analytical workflow; therefore, the reported concentrations specifically represent the fraction ≥100 μm. This methodological constraint likely leads to a conservative estimate of total MPs abundance, as the finer fraction of MPs remained beyond the limit of definitive spectroscopic confirmation in this study. Another important limitation concerns the inferential nature of the environmental interpretations. Although the identified hotspots exhibit strong spatial correspondence with anthropogenic activities such as riverine discharges, industrial clusters, and port operations, as well as with known hydrodynamic structures within this semi-enclosed basin, the proposed mechanisms remain indirectly inferred. Processes including current-mediated transport, seasonal circulation variability, and polymer-specific settling behavior are interpreted based on established regional oceanographic knowledge and previous studies rather than direct *in situ* hydrodynamic measurements or numerical simulations. Consequently, while hydrodynamic influence is suggested by the observed spatial patterns, its quantitative role in controlling MPs transport, deposition, and accumulation within offshore sediments remains insufficiently constrained. Furthermore, the ecological risk indices applied herein (PHI, PLI, and PERI) rely on polymer-level hazard classifications and do not explicitly incorporate additive-specific toxicity or degradation-related transformations, which may introduce uncertainty into ecological risk characterization.

Addressing these limitations represents an important direction for future research. Integrating harmonized field sampling campaigns with high-resolution three-dimensional hydrodynamic modeling and Lagrangian particle-tracking simulations would substantially improve the identification of dominant transport pathways, accumulation zones, and source-receptor relationships. Additionally, incorporating sediment granulometry, mineralogical composition, and organic carbon content into future investigations could help clarify polymer-specific settling dynamics and retention mechanisms, thereby improving interpretation of depositional patterns in offshore environments. The integration of these empirical and modeling approaches will be essential for advancing from primarily correlative assessments toward more mechanistic and predictive frameworks capable of supporting effective environmental management strategies.

The ecological risk evaluation based on the PLI also warrants cautious interpretation. This index relies on a study-specific baseline concentration (C_o_ᵢ), which enables internal comparison among sampling sites but inherently limits cross-regional benchmarking. Establishing standardized background concentrations for MPs in Persian Gulf sediments would significantly improve the comparability and robustness of future regional assessments. Moreover, incorporating polymer-specific degradation kinetics, additive release behavior, and long-term environmental aging processes into future risk evaluations would enhance temporal resolution and reduce uncertainty associated with ecological hazard estimation. Within this broader methodological context, it is also important to position the present risk assessment approach relative to the rapidly evolving field of MPs ecological risk evaluation. Although several advanced frameworks have been proposed to more explicitly integrate exposure dynamics, hazard characterization, and polymer-specific toxicity, their practical implementation in field-based sediment studies remains limited. The absence of harmonized toxicity thresholds, inconsistencies in exposure metrics, and the scarcity of comprehensive ecotoxicological datasets across polymer types and particle size fractions continue to constrain their application.^49^^,^^64^ These challenges are particularly pronounced in data-limited marine environments such as the offshore Persian Gulf, where baseline information on MPs abundance, polymer composition, and ecological responses remains scarce. Under these conditions, the use of established indices, namely the PHI, PLI, and PERI, provides a practical screening-level framework for evaluating MPs contamination levels and associated ecological risks in sedimentary environments. These indices benefit from wide adoption, methodological reproducibility, and a substantial body of comparative studies, allowing the present findings to be interpreted within an established global framework. As polymer-specific toxicity databases expand and more standardized experimental methodologies emerge, future studies will be better positioned to incorporate next-generation risk assessment frameworks and achieve more refined, regionally calibrated evaluations of MPs ecological risk^49^^,^^65^ Despite these limitations, the present study provides one of the first region-specific screening-level assessments of MPs contamination and associated ecological risk in offshore sediments of the Persian Gulf, thereby establishing an important baseline for future mechanistic and long-term monitoring studies.

### Conclusion

Based on the comprehensive assessment of MPs contamination in offshore sediments of the Persian Gulf, the region appears to act as a potential accumulation zone for MPs, shaped by multiple anthropogenic pressures and the basin’s semi-enclosed circulation. The spatial distribution of MPs indicates localized concentration zones; however, these patterns should be interpreted with caution due to methodological limitations, particularly the inability to reliably identify polymer types for particles smaller than 100 μm and the absence of hydrodynamic modeling. Stations exhibiting elevated ecological risk metrics, such as T1S3, highlight areas where further investigation and monitoring are warranted. The predominance of fibers (74%) and dark-colored particles (67%), together with a polymer composition dominated by PET (41%) and followed by PE (23%), PP (15%), PVC (10%), PA (6%), and PS (5%), characterizes the MPs profile observed in the study area. This composition may reflect a pollution pattern potentially associated with regional usage patterns and waste management practices. To avoid over attribution, the interpretation of color and polymer trends has been framed as indicative rather than determinative, acknowledging the modifying effects of environmental weathering, photo-oxidation, and biofouling. Polymer and color signatures cannot be conclusively linked to specific sources without integrating additive level chemistry and regional emission inventories. The integrated ecological risk assessment using PHI, PLI, and PERI revealed substantial spatial heterogeneity. While most offshore areas exhibited low pollution loads, stations closer to anthropogenic pressures displayed elevated hazard levels. However, these indices are interpreted as relative indicators, given their dependence on polymer-level hazard categories and the lack of additive-specific toxicity or polymer aging data. Accordingly, the reported risk values should be viewed as comparative metrics within the sampled domain rather than absolute measures of ecological impact. Overall, the study provides one of the first offshore sediment baselines for the central Persian Gulf, offering a framework that can support future regional assessments. The conclusions are presented within the boundaries of the dataset’s analytical constraints, including density-based extraction bias and the limited resolution for particles <100 μm, which may underrepresent the most bioavailable size fractions. These limitations underscore the need for caution when extrapolating the findings to finer sediment fractions or nearshore environments with different hydrodynamic regimes. The findings highlight the importance of sustained monitoring efforts in areas showing elevated risk and may help inform future management strategies. A multifaceted strategy remains essential, including improved waste management infrastructure, strengthened regulation of industrial effluents, and regionally coordinated monitoring programs capable of capturing temporal trends.

Future research should incorporate sediment logical parameters (e.g., grain size, TOC(total organic carbon)), *in situ* hydrodynamic measurements, and advanced 3D/Lagrangian modeling to more robustly elucidate transport pathways and depositional controls. Cross-disciplinary integration including oceanography, ecotoxicology, sedimentology, and polymer science will be critical for resolving the mechanisms governing MPs transport and retention in this semi-enclosed basin. Addressing MPs pollution in the Persian Gulf will be important for supporting ecosystem integrity and fisheries sustainability. This study emphasizes that a full understanding of MPs dynamics in the region requires integrated, multidisciplinary efforts and enhanced cross-border cooperation. Such cooperation is particularly important given the Gulf’s shared circulation patterns and the transboundary nature of plastic emissions, which collectively demand harmonized regional monitoring and mitigation frameworks. A comprehensive understanding of MPs dynamics in the Persian Gulf will ultimately require improved characterization of smaller particle fractions, enhanced temporal monitoring, and a clearer understanding of transport processes and biological interactions within the regional ecosystem.

## Resource availability

### Lead contact

Further information and requests for resources should be directed to and will be fulfilled by the lead contact, Ali Mehdinia (mehdinia@inio.ac.ir).

### Materials availability

This study did not generate any novel or unique reagents. All materials and methods used for data generation and analysis are described in the manuscript.

### Data and code availability

All data generated and reported in this study are available from the lead contact upon reasonable request. This study did not generate or report any original code. Any additional information required to reproduce or reanalyze the data presented in this article is available from the lead contact upon reasonable request.

## Acknowledgments

This work was supported by the 10.13039/501100015085Iranian National Institute for Oceanography and Atmospheric Science (10.13039/501100015085INIOAS) and the Iran National Environment Fund (INEF) (project no. 9310/1020571).

## Author contributions

P.A., data curation, formal analysis, investigation, methodology; writing – original draft, and writing -review and editing; A.S., data curation, formal analysis, and investigation; A.M., sampling, conceptualization, methodology, supervision, and writing-review and editing.

## Declaration of interests

The authors declare no competing interests.

## STAR★Methods

### Key resources table

**Table:** 

REAGENT or RESOURCE	SOURCE	IDENTIFIER
**Chemicals, peptides, and recombinant proteins**		

Extran® detergent (5%)	Merck (Darmstadt, Germany)	Chemical reagent
Hydrogen peroxide (H_2_O_2_, 30%, diluted to 3%)	Merck (Darmstadt, Germany)	Chemical reagent
Hydrochloric acid (HCl, 1 M)	Merck (Darmstadt, Germany)	Chemical reagent
Fenton reagent (FeSO4 + H_2_O_2_)	Sigma-Aldrich (Steinheim, Germany)	Chemical reagent
Saturated sodium chloride (NaCl) solution	Merck (Darmstadt, Germany)	Chemical reagent
Saturated sodium iodide (NaI) solution	Merck (Darmstadt, Germany)	Chemical reagent

**Software and algorithms**		

Google Earth Pro	Google Co. (California, USA)	Software
ArcGIS 10.3	ESRI (California, USA)	Software
Labspec 6	Horiba Scientific (Kyoto, Japan)	Software
IBM SPSS Statistics 25	SPSS Inc., USA	Software

**Other**		

R/V Persian Gulf Explorer	Iranian National Institute for Oceanography and Atmospheric Science (INIOAS), Iran	Research vessel
Van Veen grab sampler	HYDRO-BIOS (Altenholz, Germany)	Field equipment
Stainless-steel spoon (pre-cleaned)	Lab Chem Corporation (Ahmedabad, Gujarat, India)	Sampling tool
0.45 μm membrane filters	MACHEREY-NAGEL (Dueren, Germany)	Filter
Whatman GF/C glass fiber filters, 55 mm, 1.2 μm	Sigma-Aldrich (Steinheim, Germany)	Filter
needle device	Local	Instrument
OZL- 45 stereomicroscope	KERN & SOHN (GmbH, Balingen, Germany)	Instrument
LabRAM HR Evolution confocal Raman microscope	Horiba Scientific (Kyoto, Japan)	Instrument

### Experimental model and study participant details

There are no experimental models (animals, human participants, plants, microbe strains, cell lines, primary cell cultures) used in the study.

### Method details

#### Sampling design and sediment collection

A stratified random sampling design was used along the Iranian side of the Persian Gulf. The study area was divided into five environmental strata: estuarine, harbor, urban, industrial, and relatively undisturbed ecologically sensitive areas. Sampling transects were delineated using Google Earth, and sampling stations were randomly selected within each stratum along the predefined transects (Figure 1). Surface sediments were collected during the PGE 2201 research cruise, conducted from January to February 2022 aboard the R/V *Persian Gulf Explorer*, operated by the Iranian National Institute for Oceanography and Atmospheric Science (INIOAS). At each of 39 stations, three replicate surface sediment samples were collected from the upper 0–5 cm layer using a Van Veen grab sampler^36^ (*n* = 117) sediment samples in total. The three samples collected at each station represented subsamples collected from a single grab. Samples were transferred into pre-cleaned containers, and transported to the laboratory at approximately 20°C. Samples were stored at 20°C before analysis.

#### Contamination control and quality assurance

Strict contamination-control procedures were implemented throughout sample preparation and particle analysis to minimize airborne and reagent-derived contamination.^11^^,^^66^ Laboratory windows remained closed during processing, and work surfaces were cleaned with 70% ethanol before each analytical session. Glassware was rinsed with prefiltered distilled water and covered with aluminum foil^67^ whenever it was not in use. The use of plastic laboratory equipment was minimized, and laboratory personnel avoided wearing synthetic clothing during sample preparation and particle examination. All reagents and solutions (distilled water, Extran® detergent, H_2_O_2_, HCl, Fenton reagent, NaCl, and NaI) were pre-filtered through 0.45 μm membrane filters before use.^10^ Passive air samplers were placed in the laboratory to monitor background deposition.^6^ Recovery efficiency was validated by spiking microplastic-free sediment (100 g) with 10 pre-characterized polymer particles size range (0.5–2.5 mm) in triplicate (*n* = 3). Procedural blanks were processed alongside the sediment samples. For each blank, a prefiltered was exposed to the laboratory environment for the same duration as the samples and subsequently examined under the same stereomicroscopic conditions.^68^ Passive air-deposition controls were used to monitor potential airborne contamination in the laboratory during sample processing.^69^ Following exposure, the filters were covered and examined using the same visual-screening procedure applied to the samples.

#### Recovery experiment

Method recovery was evaluated using microplastic-free sediment. For this purpose, plastics particles (0.5–2.5 mm) were initially prepared after confirming their polymer type using Raman spectroscopy, and microplastic-free sediment (100 g) was prepared by manually removing all visually identifiable plastic-like particles^24^^,^^70^^,^^71^ Recovery rates were determined by spiking10 pre-characterized (via Raman spectroscopy) microplastic particles of each polymer type (sizes: 0.5–2.5 mm) into samples and separating step was implemented according to the method previously described. The full extraction workflow was performed in triplicate (*n* = 3) to ensure reproducibility.^4^ Aliquots of 100 g sediment were spiked with 10 pre-characterized polymer particles ranging from 0.5 to 2.5 mm in size. The spiked particles included polyvinyl chloride (PVC), polyethylene (PE), polypropylene (PP), polyamide (PA), polyethylene terephthalate (PET), and polystyrene (PS). The number of particles of each polymer added to each replicate was 10. Spiked sediment samples were processed following the same extraction and identification workflow used for the field-collected sediment samples.

#### Sediment preparation and chemical digestion

Approximately 200 g of each sediment sample was oven-dried at 50 °C for 48h. The dried sediment was transferred to a glass jar and mixed with 500 mL of 5% Extran® detergent solution. The sediment detergent suspension was manually stirred for 30 min to disaggregate sediment aggregates and facilitate the release of particles associated with the sediment matrix. The suspension was subsequently allowed to settle undisturbed for 24h. After settling, the resulting supernatant, potentially containing suspended microplastic particles, was carefully decanted into a clean glass beaker and subjected to chemical digestion. The collected supernatant was treated with 100 mL of H_2_O_2_ (3%), prepared by diluting a 30% H_2_O_2_ (diluted to 3% for controlled reaction) and left to react for 24 h to oxidize organic matter. The collected supernatant was treated with 10 mL of freshly prepared Fenton’s reagent (5% FeSO4 and 30% H_2_O_2_) under gentle stirring. The reagent was added gradually while the temperature was maintained below 40°C. The mixture was then allowed to react for 24 h. Following chemical digestion, the resulting suspension was subjected to sequential density separation using saturated NaCl and NaI solutions.

#### Sequential density separation

Microplastic particles were isolated using a two-step sequential density-separation procedure. In the first step, the chemically digested suspension was transferred to a clean glass separating funnel and mixed with 200 mL of saturated NaCl solution with a measured density of approximately 1.2 g cm³. This step was intended primarily to recover relatively low-density polymers, including PE and PP. The upper liquid phase containing buoyant particles was carefully transferred to a clean glass reservoir. The lower phase remaining after collection of the NaCl supernatant was retained for the second density-separation step. In the second step, the retained lower phase was mixed with 150 mL of saturated NaI solution with a measured density of approximately 1.8 g cm³. The total mixing and settling period was 24 h. The upper liquid phase was subsequently transferred to a separate clean glass reservoir. The sequential use of NaCl and NaI facilitated the recovery of polymers over an approximate density range of 0.9–1.8 g cm^−^³, including relatively high-density polymers that could not be efficiently separated using NaCl alone.

#### Vacuum filtration

The upper liquid phases collected from the NaCl and NaI density-separation steps were vacuum-filtered separately through Whatman GF/C glass-fiber filters, 55 mm in diameter, with a nominal pore size of 1.2 μm.

#### Visual screening and classification

Particles retained on the glass-fiber filters were examined using an OZL-45 stereomicroscope (KERN & SOHN GmbH, Balingen, Germany) at 7–45× magnification. Suspected microplastic particles were preliminarily selected according to their morphology, including shape, color, surface texture, uniformity of coloration, and absence of visible cellular or other biological structures. These criteria were used only for preliminary screening and were not considered definitive evidence of synthetic polymer composition. A hot-needle test was used as an additional preliminary screening procedure following Kor et al. (2020) and Mehdinia et al.^47^^,^^72^ Visual examination and hot-needle testing were used only to select suspected microplastic particles. Polymer composition was subsequently evaluated by Raman microspectroscopy. Particles smaller than 100 μm were excluded from quantitative analysis because their morphology and polymer composition could not be determined consistently under the analytical conditions used. Therefore, the operational lower size limit for particle quantification and Raman-based polymer identification was 100 μm. Particles were assigned to the following six size classes: (100 ≤ L < 500 μm, 500 ≤ L < 1000 μm, 1000 ≤ L < 1500 μm, 1500 ≤ L < 2000 μm, 2000 ≤ L < 2500 μm, 2500 ≤ L < 3000 μm). Particles larger than or equal to 3000 μm were absent. Particles were classified morphologically as fibers, films, cylinders, pellets, or fragments. Particle colors were grouped as white/transparent, black/gray, blue/green, red/pink, or yellow/orange. Classification was performed by more observers, and observer agreement was evaluated.

#### Identification and polymer characterization

##### Raman polymer identification

Suspected particles were subjected to a hot-needle test and visually characterized under a stereomicroscope (OZL-45, KERN & SOHN GmbH, Balingen, Germany; magnification range 7–45×; optical resolution ∼5–10 μm). Polymer identification was performed using a confocal Raman microscope (Horiba LabRam HR Evolution; Horiba Scientific, Japan) equipped with an integrated optical microscope. Raman spectra were acquired over the 400–3000 cm^−1^ range using a 50× objective, with an acquisition time of 20 s per particle. Three excitation lasers were applied based on particle morphology: 532 nm and 633 nm for fibrous particles, and 785 nm for granular particles. Spectral acquisition and processing were carried out using Labspec 6 software. The acquired spectra were compared against the internal Labspec 6 reference library as well as an external reference library provided by Dr. Freek Ariese. Polymer assignment was based on spectral concordance of characteristic Raman peak positions and relative intensity patterns, considering the absence of significant spectral interferences. A particle was classified as a polymer when the spectral match coefficient was ≥0.80, and the assignment was further validated by visual inspection of key diagnostic peaks. Although particles smaller than 100 μm could be visually observed during microscopic examination, they were excluded from the final polymer-type determination because their Raman spectra did not consistently meet this confidence threshold. Accordingly, only particles ≥100 μm were included in the final Raman-based polymer identification. In total, 103 representative particles were analyzed by Raman, of which 93 particles (approximately 90%) were confirmed as synthetic polymers.

### Quantification and statistical analysis

#### Microplastic risk indices

##### Polymer Hazard Index (PHI)

The Polymer Hazard Index (PHI) was employed for this purpose, and was calculated according to the below standard formulation proposed by Mathew et al. (2024): PHI = ∑Pn×Sn,^73^ Where PHI quantifies the hazard associated with the polymers present in MPs, Pn represents the relative fraction of each polymer type expressed as the number of particles of that polymer divided by the total number of MPs detected at each sampling station. This ensures that PHI is station-specific and reflects localized risk levels. Sn denotes the hazard rating assigned to each polymer type, based on toxicological risk,^19^^,^^74^ propensity for additive leaching, and affinity for contaminant adsorption, as reported in Mathew et al.^73^ The polymer hazard scores ultimately originate from the hazard ranking framework proposed by Lithner et al.^19^ which evaluates polymers based on the toxicological classifications of their constituent monomers. In this study, the hazard scores assigned to each polymer type polyethylene (PE), polypropylene (PP), polyethylene terephthalate (PET), polystyrene (PS), polyvinyl chloride (PVC), and polyamide (PA) are 11, 1, 4, 30, 10,001 and 47, respectively.

#### Pollution Load Index (PLI)

The PLI for each sampling site was calculated based on the concentration factors (${\text{CF}}_{i}$), defined as follows:^48^

$$CF_{i}=\sqrt{\frac{C_{i}}{C_{oi}}},PLI=\sqrt{CF_{i}}$$

The square-root formulation is adopted from Zhang et al.^7^ to reduce data dispersion and stabilize variability across stations.^75^ The Pollution Load Index for each site was then computed. Where $C_{i}$ is the concentration of microplastics at each sampling location, and $C_{\text{oi}}$ represents the baseline concentration of microplastics, was defined as the minimum observed MP concentration among all sampling sites, serving as an intra-study reference baseline in the absence of validated pre-industrial background data.^48^^,^^55^^,^^76^

#### Potential Ecological Risk Index (PERI)

Ecological risk factor, $E_{r}^{i}$ was then determined using the following formula:

$$C_{f}^{i}=\frac{C^{i}}{C_{n}^{i}},T_{r}^{i}=\sum_{n=1}^{n}\left(\frac{Pn}{Ci}\times Sn\right),E_{r}^{i}=T_{r}^{i}\times C_{f}^{i}$$

This corrected formulation ensures that the toxicity coefficient (Tᵣᶦ) reflects polymer-specific chemical hazards rather than the previous term ($\frac{\text{Pn}}{\text{Ci}}\times \text{Sn}$), which could distort risk contributions and is not supported by established ecological risk frameworks.^26^ The cumulative risk posed by individual polymer types contributes to the overall Potential Ecological Risk Index (PERI). PERI values below 150 imply negligible ecological risk, while values between 150 and 300 represent moderate risk. PERI values from 300 to 600 suggest serious risk (considerable ecological concern); 600–1200 indicate high ecological risk^77^; and values exceeding 1200 are associated with extremely severe ecological threats (Table S2).^5^^,^^10^^,^^78^

#### Statistical analysis

Statistical analyses were performed using IBM SPSS Statistics 25 software (SPSS Inc., USA). Following data entry and sorting by sampling stations, descriptive statistics were calculated. The normality of data distribution was assessed using the Shapiro-Wilk test. As the data violated the assumption of normality, non-parametric tests were employed. The Kruskal-Wallis H test was used to compare overall differences among sampling stations, and post-hoc pairwise comparisons were conducted using the Mann-Whitney U-test. A Bonferroni correction was applied to control Type I error associated with multiple comparisons, and statistical significance was set at α = 0.05.

### Additional resources

This study did not generate or contribute to a new website or forum, and it was not part of a clinical trial.
