# Supplementary material for: Microplastics in Persian Gulf offshore sediments: Distribution, polymer composition, and ecological risk assessment
Source: iScience. 2026 Aug 28;29(9):117263. doi: 10.1016/j.isci.2026.117263 (PMC13545467; doi:10.1016/j.isci.2026.117263)
Supplement: Supplementary file 1 [file mmc1.pdf]

iScience, Volume *29*

Supplemental information

Microplastics in Persian Gulf offshore sediments: Distribution, polymer composition, and ecological risk assessment

Parisa Amiri, Afsaneh Shahbazi, and Ali Mehdinia

Figure Caption

Figure 1S. Microplastic Size of the Persian Gulf Sediments.

Figure 1S.1. Microplastic Size of the Persian Gulf Sediments by sampling station.

Figure 2S. Microplastic Color of the Persian Gulf Sediments.

Figure 2S.1. Microplastic Color of the Persian Gulf Sediments by sampling station.

Figure 3S. Microplastic Shape of the Persian Gulf Sediments.

Figure 3S.1. Microplastic Shape of the Persian Gulf Sediments by sampling station.

Figure 4S. Polymer type of MPs in the Persian Gulf.

Figure 4S.1. Polymer type of MPs in the Persian Gulf by sampling station.

Table 1S. Descriptive Statistics of the Data's Persian Gulf Offshore Sediments.

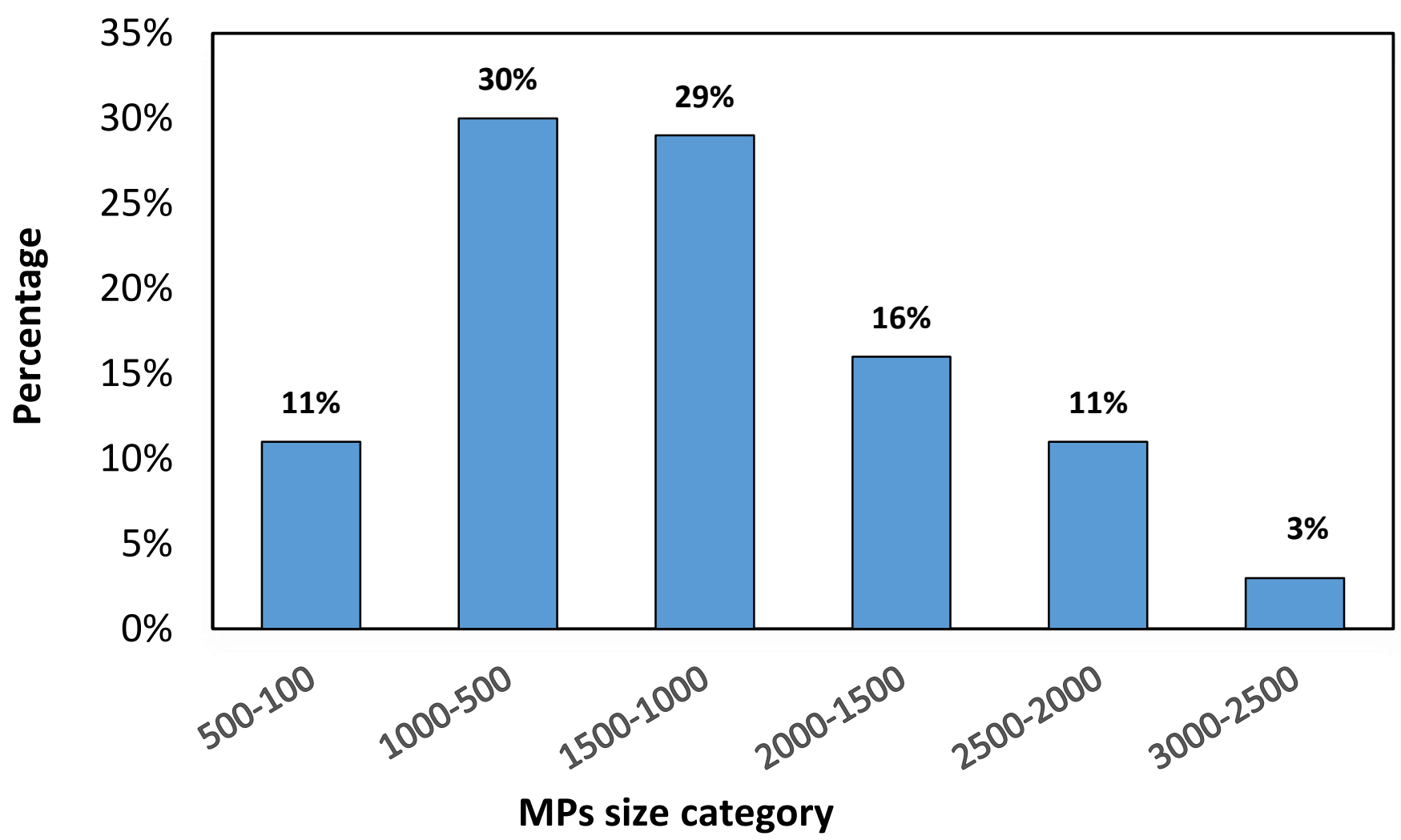

Figure 1S. Microplastic Size of the Persian Gulf Sediments

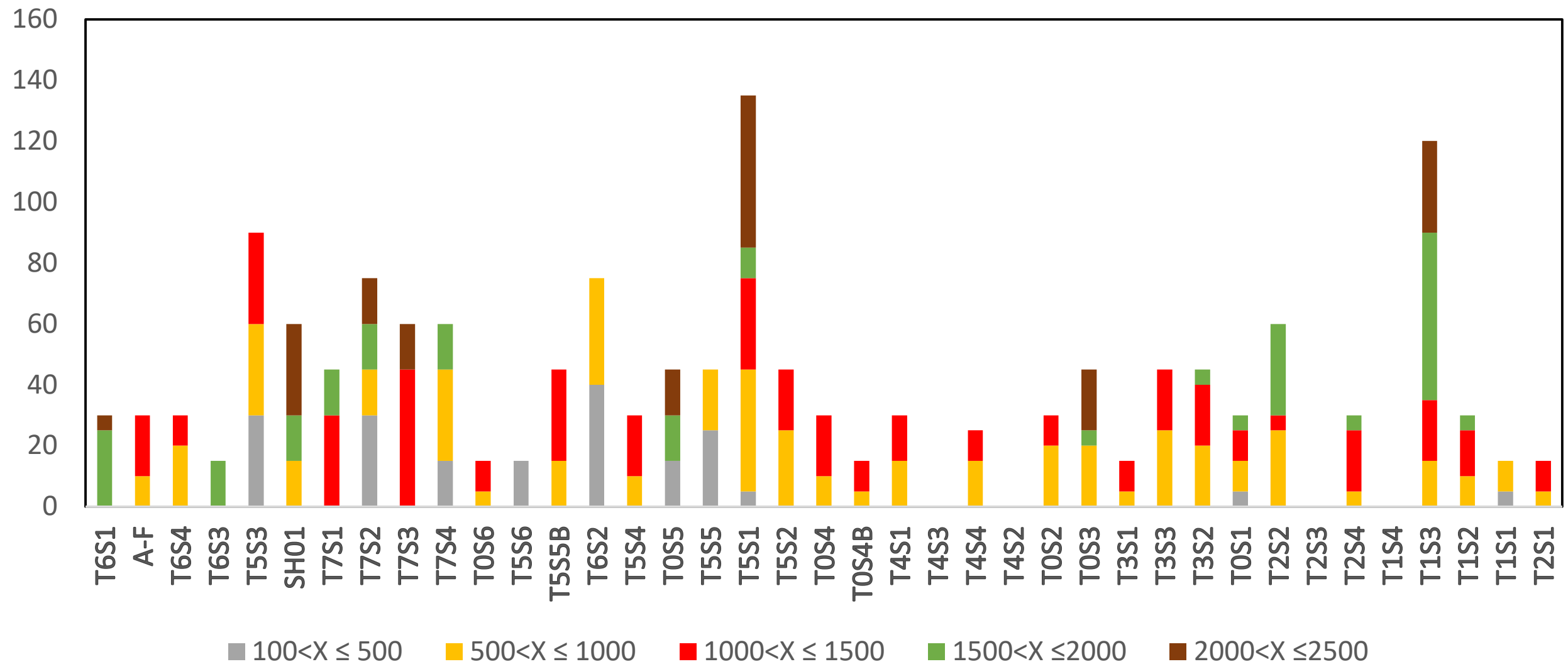

Figure 1S.1. Microplastic Size of the Persian Gulf Sediments by sampling station

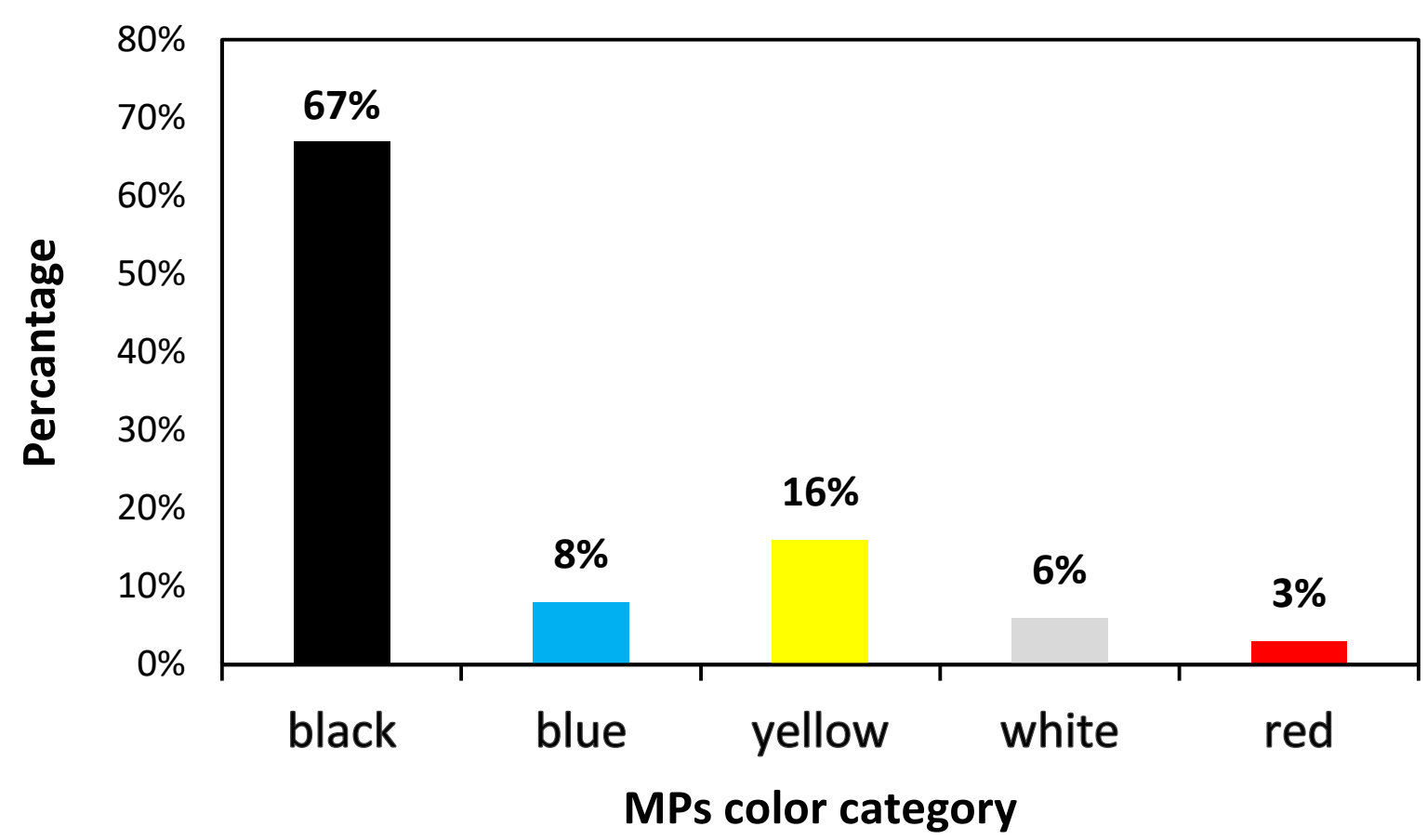

Figure 2S. Microplastic Color of the Persian Gulf Sediments

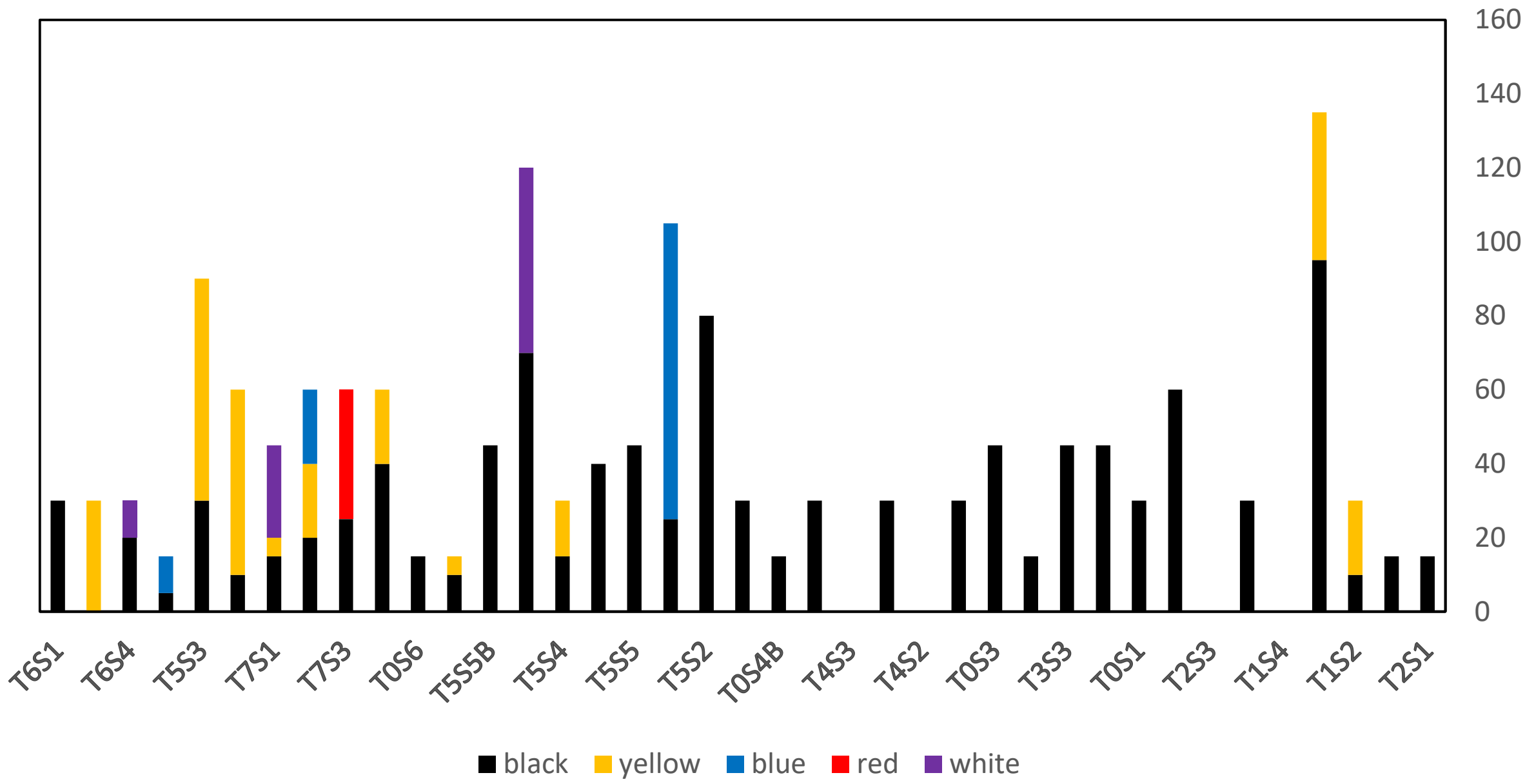

Figure 2S.1. Microplastic Color of the Persian Gulf Sediments by sampling station

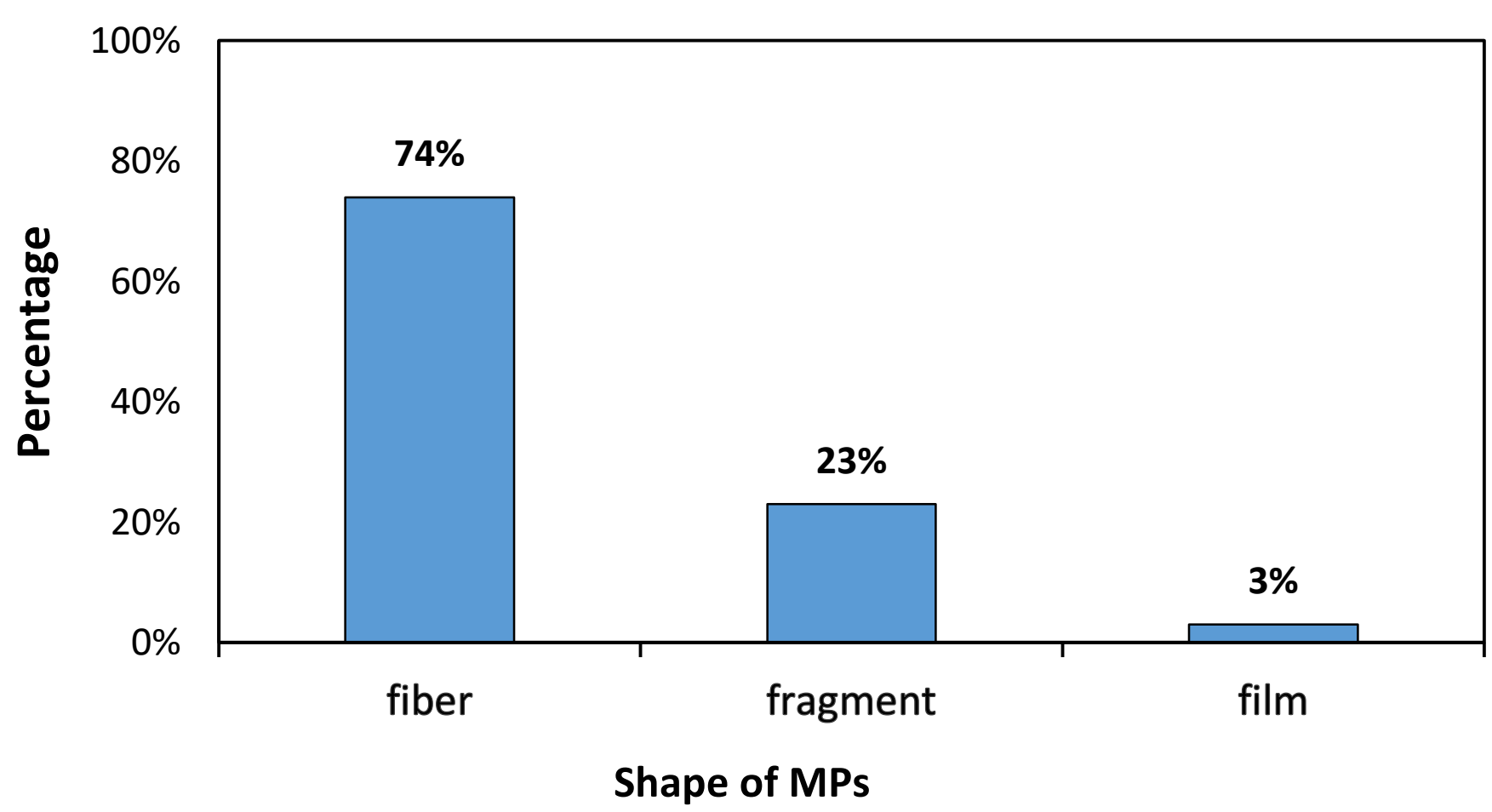

Figure 3S. Microplastic Shape of the Persian Gulf Sediments

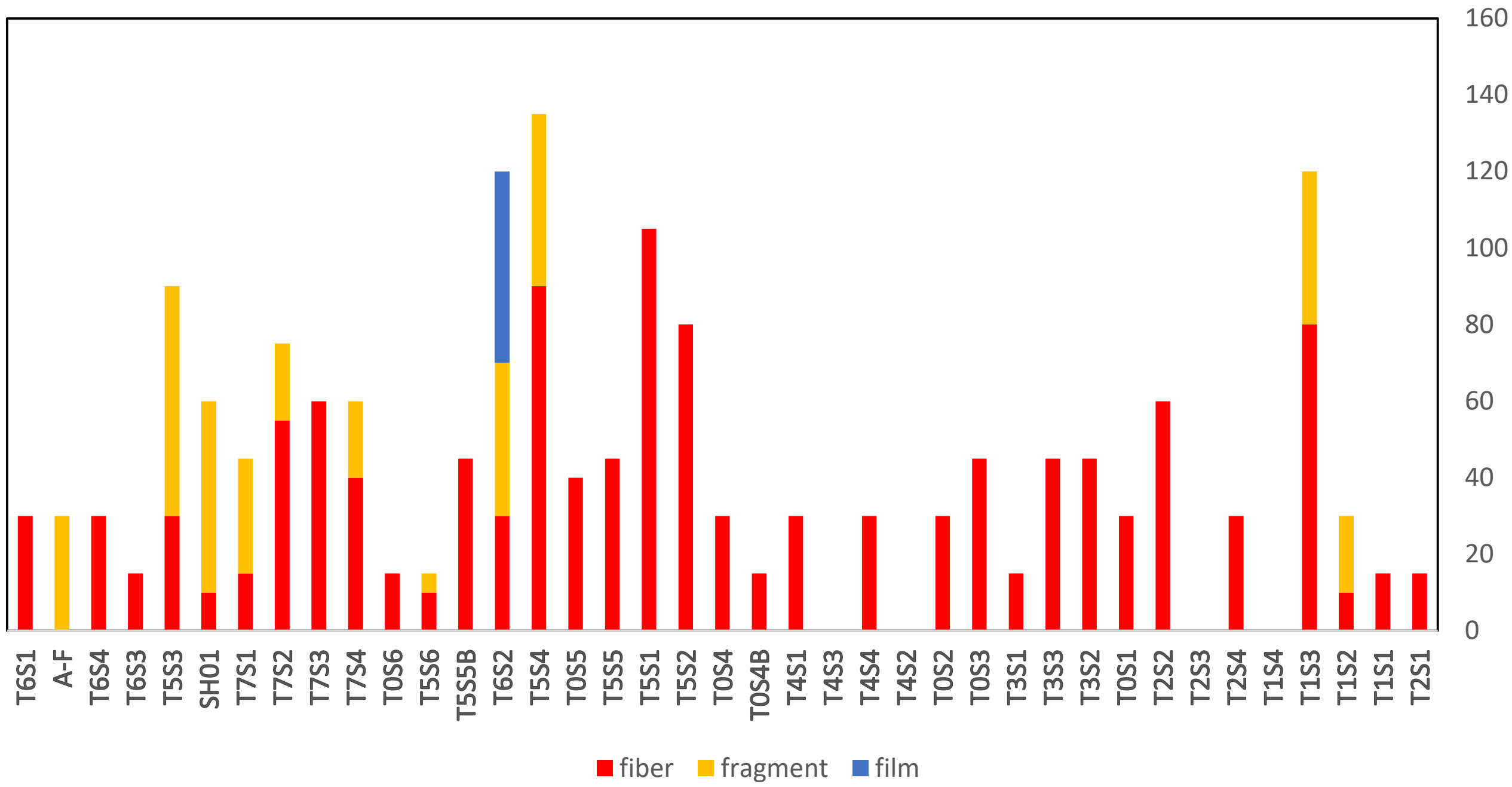

Figure 3S.1. Microplastic Shape of the Persian Gulf Sediments by sampling station

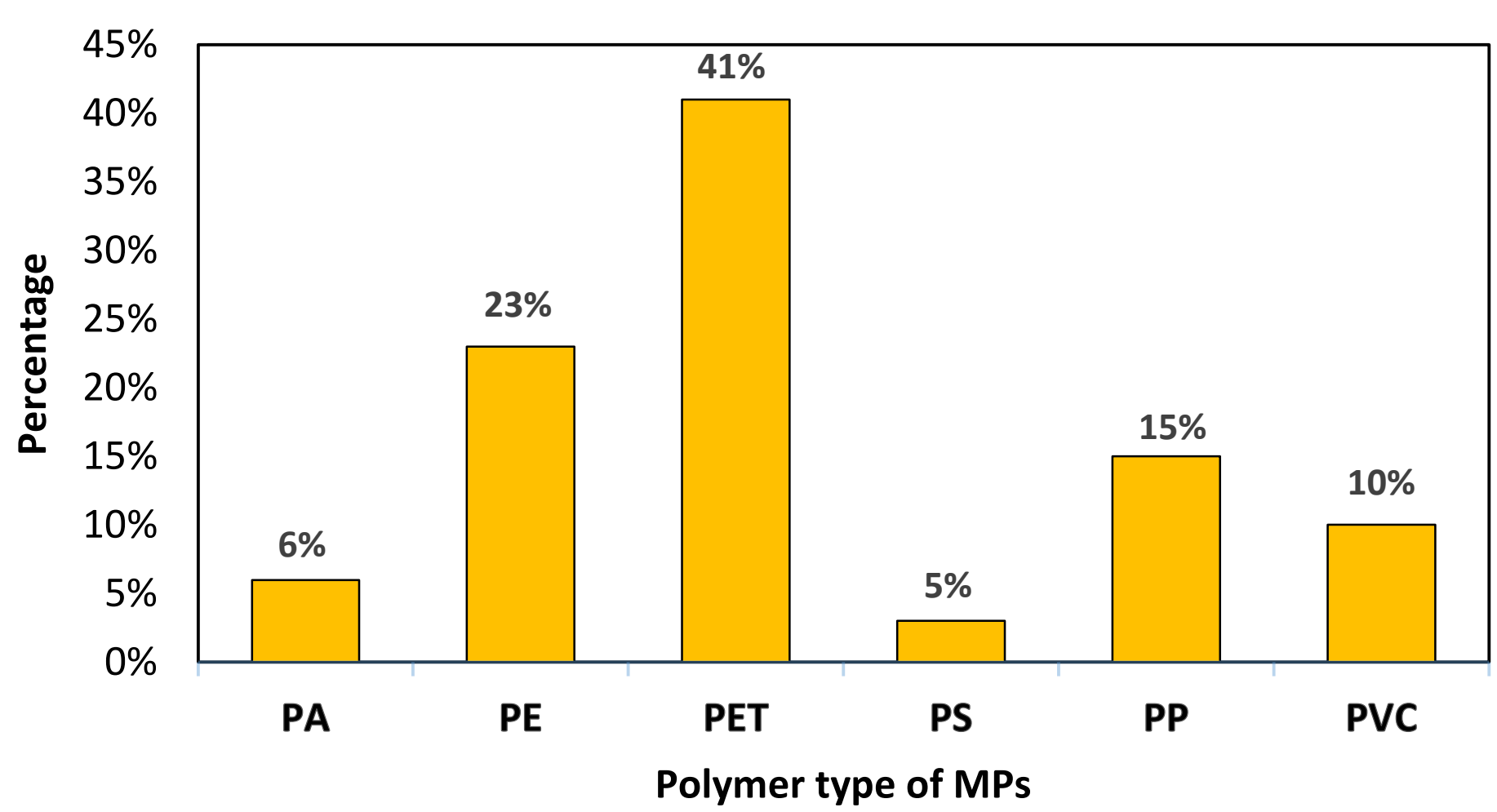

Figure 4S. Polymer type of MPs in the Persian Gulf

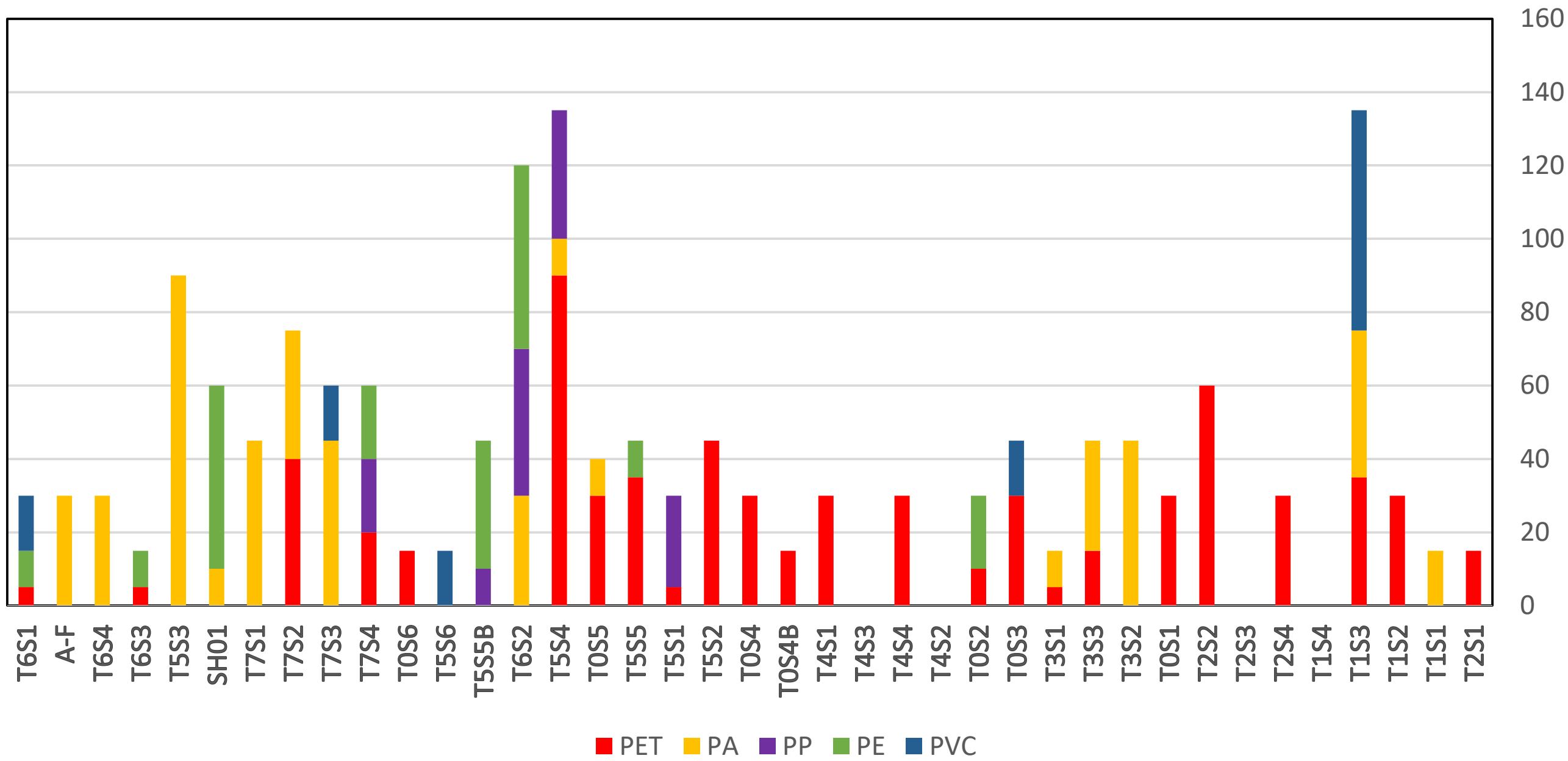

Figure 4S.1. Polymer type of MPs in the Persian Gulf by sampling station

Table 1S. Descriptive Statistics of the Data's Persian Gulf Offshore Sediments

ST	Mean ± Standard Deviation	ST	Mean ± Standard Deviation
**T2S1**	5 ± 5	**T5S5**	15 ± 5
**T1S1**	5 ±5	**T0S5**	15 ± 0
**T1S2**	10± 0	**T5S1**	10± 5
**T1S3**	40 ± 17.32	**T6S2**	40 ± 10
**T2S4**	10 ± 5	**T5S5B**	15 ± 5
**T2S2**	20 ± 8.66	**T5S6**	5 ± 0
**T0S1**	10 ± 13.22	**T0S6**	5 ± 5
**T3S3**	15 ± 5	**T7S4**	20 ± 0
**T3S2**	15 ± 0	**T7S3**	20 ± 13.22
**T3S1**	5 ± 0	**T7S2**	25 ± 8.66
**T0S3**	15 ± 0	**T7S1**	15 ± 10
**T0S2**	10 ± 0	**SH01**	20 ± 10
**T4S4**	10 ± 8.66	**T5S3**	30 ± 10

**T4S1**	10 ± 13.22	**T6S3**	5 ± 5
**T0S4B**	5 ± 8.66	**T6S4**	10 ± 0
**T0S4**	10 ± 5	**A-F**	10 ± 0
**T5S2**	15 ± 5	**T6S1**	10 ± 5
**T5S4**	45 ± 10		

Table 2S: **Criteria for assessing risk levels of Microplastic pollution and corresponding ecological risk classification in sediment samples.** [40]

PHI		PLI		PERI	
**Criteria**	**Hazard category**	**Criteria**	**Hazard category**	**Criteria**	**Risk category**
**0-1**	**1**	**<10**	**1**	**<150**	**Minor**
**1-10**	**2**	**10-20**	**2**	**150-300**	**Medium**
**10-100**	**3**	**20-30**	**3**	**300-600**	**High**
**100-1000**	**4**	**>30**	**4**	**600-1200**	**Danger**
**>1000**	**5**	**-**	**-**	**>1200**	**Extreme danger**
